# Predictors of Ultrasound-Derived Muscle Thickness and Echo Intensity After Acute Incomplete Spinal Cord Injury During Inpatient Rehabilitation: An Exploratory Observational Cohort Study

**DOI:** 10.3390/jcm15041570

**Published:** 2026-02-16

**Authors:** Matthew Rong Jie Tay, Keng He Kong

**Affiliations:** 1 Institute of Rehabilitation Excellence (IREx), Tan Tock Seng Hospital Rehabilitation Centre, Singapore 308433, Singapore; 2 Lee Kong Chian School of Medicine, Nanyang Technological University, 11 Mandalay Road, Singapore 308232, Singapore

**Keywords:** ultrasonography, skeletal muscle, quadriceps muscle, spinal cord injuries, neurologic disorders, rehabilitation

## Abstract

**Background/Objectives:** Muscle wasting is often observed in the acute phase after spinal cord injury (SCI). We aim to investigate the factors determining rectus femoris muscle thickness and echo intensity on discharge for patients who had acute incomplete spinal cord injury undergoing inpatient rehabilitation. **Methods:** This is a prospective exploratory observational cohort study, conducted in a standalone inpatient multi-specialty tertiary rehabilitation center in Singapore. Forty-five patients with incomplete SCI, defined as American Spinal Injury Association Impairment Scale (AIS) B–D were recruited from January 2020 to October 2021. Variables including clinico-demographic data, lower limb spasticity, Lower Extremity Muscle Score (LEMS), Functional Independence Measure (FIM) motor score on admission were collected. Muscle ultrasound of the rectus femoris thickness and echo intensity were obtained at 6 weeks after acute SCI via standardized protocols. Stepwise multiple regression analyses were performed to identify the factors that were significant for rectus femoris muscle thickness and echo intensity on discharge. **Results:** The mean age of participants was 59.6 ± 16.6 years, with patients having AIS of B (11.1%), C (28.9%) or D (60.0%). Rectus femoris muscle thickness on discharge had a significant association with body mass index (B = 4.62; CI = 1.77, 7.47; *p* = 0.002) and onset of mobilization (B = −4.97; CI = −9.46, −0.484; *p* = 0.031). The significant variables associated with rectus femoris echo intensity on discharge were age (B = 0.546; CI = 0.126, 0.967; *p* = 0.012) and onset of mobilization (B = 2.49; CI = 0.439, 4.53; *p* = 0.019). **Conclusions:** Our findings suggest that age, body mass index and a delayed onset of mobilization may have significant impact on muscle ultrasound parameters. Patients with incomplete SCI may benefit from early mobilization and nutritional assessment for improved muscle strength and function.

## 1. Introduction

Muscle wasting is often observed in the acute phase after spinal cord injury, and is a contributing factor in muscle weakness. The immediate loss of voluntary muscle activity due to SCI leads to a disruption in normal muscle homeostasis, resulting in both a decrease in muscle fiber size and an increase in muscle catabolism [1]. Chronic inflammatory responses and oxidative stress in muscles further compounds muscle loss [1]. This can lead to marked muscle atrophy, with the average muscle cross-sectional area of the quadriceps being reported to be reduced by 16% in the first 6 months after complete SCI [2]. While muscle wasting is more pronounced in individuals with complete SCI, those with incomplete SCI are also affected by muscle atrophy. The degree of atrophy depends on the extent of the injury, the level of functional preservation, and the extent of motor neuron involvement. Notably, even partial loss of spinal cord function leads to significant disruptions in motor control and muscle function. In incomplete SCI patients, muscle CSA has been found to be reduced as well when compared to healthy volunteers at 6 weeks post injury [3].

The accumulation of fat within muscle tissue is a well-established feature of muscle atrophy, and it has been linked to impaired insulin sensitivity and metabolic dysfunction [4]. This phenomenon has been reported in the acute phase following injury, where intramuscular fat levels are reported to be up to three times higher in incomplete SCI patients compared to healthy controls [4]. Muscle atrophy and increased fatty infiltration of muscle, in turn, has been shown to be negatively associated with functional outcomes in acute spinal cord injury [5]. Echo intensity, as measured on ultrasound, reflects muscle quality by detecting intramuscular fat and fibrous tissue infiltration [6,7].

Muscle wasting, which is characterized by a reduction in the number and/or size of muscle fibers, is influenced by denervation due to injury to motor neurons, as well as disuse due to decreased muscle loading [8,9,10,11]. Various modalities, including early mobilization and electrical stimulation, have been shown to increase muscle area and lean muscle mass, with a study reporting increased muscle cross sectional area ranging from 3.8 to 56.9% on MRI, in patients with acute SCI injury after early mobilization intervention [12]. Additionally, various impairment and functional scales in SCI have been shown to have correlations with muscle function and strength [13,14,15,16]. B-mode ultrasonography has emerged as a reliable, non-invasive tool for quantifying these changes, where muscle thickness serves as a proxy for muscle mass [17]. However, a specific knowledge gap remains regarding the factors predicting lower limb muscle ultrasound parameters specifically in the acute phase of incomplete SCI during inpatient rehabilitation.

We therefore aim to investigate the factors determining rectus femoris muscle thickness and echo intensity on discharge in patients with acute incomplete spinal cord injury.

## 2. Materials and Methods

### 2.1. Participants

This investigation employed a prospective single-center cohort observational exploratory design, enrolling 45 consecutive individuals with incomplete spinal cord injuries who were admitted to the spinal cord injury ward at Tan Tock Seng Hospital Rehabilitation Center between January 2020 and October 2021. Participants were approached for recruitment upon admission. Ethical approval was obtained from the NHG Domain Specific Review Board, and written informed consent was secured from all participants.

Study eligibility required participants to meet several criteria: initial acute spinal cord injury episode, injury sustained <1 month, minimum age of 21 years, pre-injury ambulatory independence, spinal cord lesion located above the second lumbar vertebra as confirmed through neurological assessment, and incomplete injury classification of American Spinal Injury Association Impairment Scale (AIS) B, C, or D.

Exclusion criteria encompassed active malignant disease, pre-existing lower extremity musculoskeletal disorders including contractures, fractures, or surgical history, concurrent active neurological disorders, and cognitive deficits that would impair comprehension of study protocols.

Tan Tock Seng Hospital Rehabilitation Center operates as a tertiary-level rehabilitation institution, delivering comprehensive residential rehabilitation programs for individuals transferred directly from acute spinal cord injury departments of partner hospitals. All spinal cord injury patients received standardized residential rehabilitation interventions consisting of two hours daily treatment for five days weekly. This program included separate one-hour sessions of physiotherapy and occupational therapy daily, incorporating mobilization techniques, ambulation training, and traditional rehabilitation approaches. No participants received neuromuscular electrical stimulation or functional electrical stimulation interventions. Mobilization timing was individualized based on rehabilitation team clinical assessment.

### 2.2. Predictor Variables

Spasticity assessment in the knee extensors and ankle plantarflexors utilized the 6-point Modified Ashworth Scale (MAS), spanning from 0 (absence of increased muscle tone) to 4 (complete rigidity in flexion or extension) [18]. To facilitate statistical analysis, ratings of 1+ were assigned a numerical value of 1.5 to preserve equal intervals [19].

Lower extremity muscle strength was evaluated using the Lower Extremity Muscle Score (LEMS), conducted within 72 h post-injury following the standardized AIS neurological assessment protocol. Assessment involved testing voluntary muscle strength across five key muscle groups (hip flexors, knee extensors, ankle dorsiflexors, long toe extensors, and ankle plantarflexors) bilaterally [20]. Individual muscle groups received scores ranging from 0 to 5 based on voluntary contraction strength. Total LEMS scores ranged from 0 to 50 points.

The Functional Independence Measure (FIM) represents a widely recognized disability assessment tool [21]. The motor component of the FIM was employed to evaluate participants’ Activities of Daily Living capabilities within 48 h of rehabilitation admission. Motor FIM scores span from 13 (complete dependence) to 91 (full independence without adaptations).

Ambulatory function was quantified using the Walking Index for Spinal Cord Injury II (WISCI II) within 48 h of rehabilitation admission. The WISCI II evaluates the level of physical assistance (personnel requirements) and assistive equipment (mobility aids) necessary for a patient to traverse 10 m, utilizing a 0–20 scale where higher scores reflect reduced functional impairment [22].

### 2.3. Ultrasound Analysis

Ultrasound assessment was performed at approximately 6 weeks after acute spinal cord injury.

Rectus femoris thickness and echo intensity measurements were obtained through B-mode ultrasonography using a Terason t3200 system (Terason Ultrasound, Burlington, MA) equipped with a 15-4 MHz linear array transducer. Image acquisition followed established protocols from previous research [23,24]. Participants were positioned supine on an examination table with lower limbs in complete extension and musculature in a relaxed state. The ultrasound probe was positioned perpendicular to the skin surface without applying pressure, with all imaging conducted in the transverse orientation. Rectus femoris assessment occurred at the muscle’s midpoint, determined as the halfway point between the anterior superior iliac spine and the inferior patellar border [25].

Image analysis was conducted using ImageJ software (National Institute of Health, Bethesda, MD, USA, version 1.46). Muscle thickness was quantified as the linear distance spanning from the superior aspect of the subcutaneous fascia to the deep aponeurotic layer [26]. Echo intensity evaluation utilized gray scale analysis, reported in arbitrary units (a.u.) through ImageJ software. A rectangular region of interest was delineated to encompass the maximum possible muscle area while excluding visible fascial planes and osseous structures. Bilateral rectus femoris ultrasonographic assessments were conducted twice to generate mean values, with the bilateral readings averaged to a single value (per patient) employed for final statistical analysis. A single operator with over five years of musculoskeletal ultrasound expertise acquired all images to ensure consistency.

### 2.4. Statistical Analysis

Sociodemographic and clinical characteristics were summarized using appropriate descriptive statistics. Spearman’s correlation analysis was employed to examine relationships between rectus femoris muscle thickness and echo intensity with the following variables: age, AIS, Body Mass Index (BMI), knee extensor spasticity, ankle plantarflexor spasticity, mobilization onset timing, FIM motor subscore, LEMS, and WISCI II. Stepwise multiple regression modeling was conducted to determine statistically significant predictive factors. The stepwise criteria for entry was *p* < 0.05 and removal was *p* > 0.10 [27]. All statistical computations were executed using Statistical Package for the Social Sciences version 26.0 (IBM Corp., Armonk, NY, USA). Two-tailed hypothesis testing was applied with a 5% significance threshold for all analyses. A sample size of 45 was determined a priori based on a power analysis for multiple linear regression (F-test). Using rectus femoris muscle thickness as the primary outcome and accounting for 7 independent predictors, we estimated that a sample size of 45 participants would provide 90% statistical power to detect a large effect size of 0.50 at a significance level of α = 0.05. These effect size assumptions were derived from internal pilot data. There was no missing data. This study was reported in accordance with the Strengthening the Reporting of Observational Studies in Epidemiology (STROBE) guidelines for cohort studies (Table S1).

## 3. Results

There were 45 patients recruited, with a mean age of 59.6 (16.6) years. Study participants had SCI of AIS B (11.1%), C (28.9%) or D (60.0%). The majority of patients had SCI from a fall (66.7%) or motor vehicle accident (22.2%). The average BMI was 23.0 (5.62) (Table 1).

Correlation coefficients examining the relationships between rectus femoris thickness or echo intensity and each study variable are displayed in Table 2. The rectus femoris muscle thickness on discharge were significantly positively associated with a higher AIS classification (Spearman’s rho = 0.407), BMI (Spearman’s rho = 0.594), FIM motor subscore on admission (Spearman’s rho = 0.361), LEMS on admission (Spearman’s rho = 0.387), WISCI II on admission (Spearman’s rho = 0.366) and negatively associated with onset of mobilization (Spearman’s rho = −0.484).

The rectus femoris echo intensity on discharge were significantly positively associated with age (Spearman’s rho = 0.351), BMI (Spearman’s rho = −0.460) and negatively associated with onset of mobilization (Spearman’s rho = −0.493).

Table 3 reports the findings from the stepwise multiple regression analyses. Multicollinearity was not detected among the independent variables within the stepwise multiple regression models. The significant variables associated with rectus femoris muscle thickness on discharge were BMI (B = 4.62; CI = 1.77, 7.47; *p* = 0.002) and onset of mobilization (B = −4.97; CI = −9.46, −0.484; *p* = 0.031). The significant variables associated with rectus femoris echo intensity on discharge were age (B = 0.546; CI = 0.126, 0.967; *p* = 0.012) and onset of mobilization (B = 2.49; CI = 0.439, 4.53; *p* = 0.019).

## 4. Discussion

We found muscle thickness on discharge was positively correlated with a higher BMI and negatively correlated with delayed onset of mobilization. An increased echo intensity was also positively correlated with age, and delayed onset of mobilization.

In our analysis, we observed that certain variables significant in univariate correlations—such as AIS, LEMS, and FIM motor scores—were not retained in the final multivariate models. This divergence is expected in clinical datasets where clinical and functional parameters often overlap. By employing a stepwise multivariate regression, we were able to filter out these shared variances to identify BMI, age, and onset of mobilization as the primary independent predictors.

Considerable atrophy of skeletal muscle is believed to occur within the first 6 weeks post acute SCI [3]. Although evidence has largely focused on exercise interventions in chronic SCI, emerging evidence highlights the role of early weight bearing in mitigating muscle atrophy and promoting recovery of function in acute SCI patients. Animal models have suggested that partial weight bearing should begin early after SCI for optimal adaptive muscular plasticity [28,29], and a systemic review found that thigh and calf muscle CSA were increased in individuals who underwent early weight bearing [30].

These results suggest an association between earlier functional loading of the muscles and preserved muscle thickness [31], which translates to increased muscle strength to support weight. Supporting this mechanism is a study by Nozoe et al., which found that quadriceps muscle thickness loss was less severe in patients with acute subarachnoid hemorrhage who received early mobilization compared against those with non-early mobilization [32]. While our findings show that an earlier onset of mobilization is associated with larger muscle mass, the observational nature of this study precludes a definitive conclusion that early mobilization directly prevents these changes.

Concerning BMI, we observe that the mechanical loading imposed by increased body weight is associated with greater muscle thickness among acute SCI patients. This may suggest that body weight functions as a stimulus for muscle architectural adaptations [33]. This mechanism is seen in healthy populations, where greater body weight provides increased mechanical loading during weight-bearing activities, leading to adaptive responses in muscle size and strength. Supporting this view are findings that a lower BMI is conversely associated with reduced muscle mass in chronic stroke [34] and SCI survivors [35]. However, this interpretation must be considered within the unique physiological context of acute incomplete SCI. Unlike healthy individuals, SCI patients experience rapid changes in body composition, including muscle atrophy and altered fat distribution. In the acute phase following SCI, higher BMI may reflect pre-injury muscle mass that has not yet undergone significant atrophy, rather than indicating current muscle health or loading capacity.

We also found age and a delayed onset of mobilization to be correlated with increased echo intensity of the rectus femoris. In healthy individuals, aging has been shown to lead to increased intramuscular fat and decreased motor function [36]. Additionally, increased intramuscular fat, as evaluated by ultrasound, has demonstrated an inverse relationship with muscle strength in older individuals [37]. Comparable observations have been documented in stroke populations, demonstrating an inverse relationship between elevated muscle echo intensity and muscle strength in both affected and unaffected extremities [38,39,40].

This suggests that older individuals may be more susceptible to muscle atrophy, indicated by an increase in intramuscular fat or fibrosis, which can be captured by echo intensity imaging. This finding is consistent with studies reporting that aging exacerbates the loss of muscle quality, including changes in muscle composition that lead to an increased proportion of fat infiltration [41]. Therefore, older individuals with SCI may be at higher risk for muscle degeneration, highlighting the need for targeted interventions aimed at preserving muscle quality in this population.

A delayed onset of mobilization emerged as a significant negative predictor of muscle thickness, with delayed mobilization correlating with greater muscle loss. This finding underscores the importance of early mobilization following SCI to prevent muscle wasting. Delayed mobilization likely exacerbates the loss of muscle mass, as muscle atrophy is known to progress rapidly during the acute phase of SCI [42]. In patients with chronic SCI, physical activity has been found to be associated with reduced fatty infiltration of lower extremity muscles [35]. Hence, delayed or insufficient mobilization may facilitate the accumulation of fatty infiltrates within muscle tissue, leading to higher echo intensity values. Early mobilization, therefore, not only helps preserve muscle mass but also contributes to maintaining muscle quality and function.

Our data suggest that age, BMI, and mobilization timing have associations with muscle status, and may potentially inform individualized rehabilitation planning. These parameters may be useful for clinicians to identify high-risk phenotypes who may require prioritized, early mobilization and targeted nutritional-functional strategies to mitigate rapid atrophy [43].

This study has several limitations worth highlighting. First, this is an exploratory study with a small sample size, which can lead to the risk of overfitting and selection bias. Second, while we identified certain predictors, our multivariate model did not adjust for key clinical variables such as patient comorbidities (e.g., diabetes or cardiovascular disease) and the use of specific medications (e.g., corticosteroids), which may independently influence muscle mass and quality. Third, there was no standardization or quantification of rehabilitation intensity; the time to mobilization and specific modalities were left to the discretion of the rehabilitation team. We were also unable to quantify the duration and use of specific mobility equipment, such as robotic or exoskeleton-assisted devices, used for each patient. Fourth, although nutritional parameters have been implicated as a factor in muscle ultrasound parameters [44], we did not investigate this relationship in depth. Fifth, while BMI was included as a proxy for mechanical loading, it is a limited measure in the SCI population as it fails to distinguish between lean mass and adiposity, and we also did not use other modalities e.g., DEXA to further quantify the lean mass index [45]. Sixth, a significant limitation is the lack of baseline ultrasound measurements. Because assessments were only performed at a single time point (approximately 6 weeks post-injury), we cannot define the trajectory of muscle changes occurring during the acute phase of recovery. Seventh, we did not perform formal intra-rater or inter-rater reliability testing for the ultrasound measurements. While a standardized protocol was followed to minimize measurement error, the absence of reliability data should be considered when interpreting the precision of our values. Lastly, the generalizability of our findings is restricted by the single-center nature and the specific cohort characteristics of this study, and larger prospective studies are needed to confirm whether early mobilization directly improves muscle ultrasound parameters in SCI patients.

## 5. Conclusions

Our findings suggest that a delayed onset of mobilization is associated with reduced muscle thickness and increased echo intensity, while a lower BMI and an older age were associated with reduced muscle thickness and increased echo intensity. These muscle ultrasound parameters may reflect the influence of these factors on muscle strength in the acute SCI period. Identification of these factors may enable rehabilitation programs to have a tailored approach for early mobilization and exercise intervention to improve muscle mass and function in the acute incomplete SCI population.

## Figures and Tables

**Table 1:** Characteristics of study participants.

Variable	*n* = 45
Age, years, mean ± SD	59.6 ± 16.6
Sex (Male: female)	33:12
Ethnicity, *n* (%)	
- Chinese	35 (77.8)
- Malay	8 (17.8)
- Indian	2 (4.4)
Etiology, *n* (%)	
- Fall	30 (66.7)
- Motor vehicle accident	10 (22.2)
- Infectious	1 (2.2)
- Inflammatory	2 (4.4)
- Vascular	2 (4.4)
AIS, *n* (%)	
- B	5 (11.1)
- C	13 (28.9)
- D	27 (60.0)
Time to ultrasound measurement from injury, weeks, mean ± SD	6.42 ± 0.336
Height, cm, mean ± SD	1.65 ± 0.0879
Weight, kg, mean ± SD	63.0 ± 17.9
Body Mass Index, mean ± SD	23.0 ± 5.62
Knee extensor spasticity on admission, MAS, mean ± SD	1.70 ± 0.558
Ankle plantarflexor spasticity on admission, MAS, mean ± SD	1.71 ± 0.528
Onset of mobilization, days, mean ± SD	5.4 ± 3.53
FIM motor subscore on admission, mean ± SD	30.4 ± 15.2
LEMS on admission, mean ± SD	27.5 ± 16.8
WISCI II on admission, mean ± SD	3.98 ± 5.72

AIS: American Spinal Injury Association Impairment Scale; MAS: Modified Ashworth Scale, FIM: Functional Independence Measure; LEMS: Lower Extremity Muscle Score; WISCI II: Walking Index for Spinal Cord Injury II.

**Table 2:** Correlation coefficients between predictor variables and rectus femoris muscle thickness, echo intensity.

Variable	Correlation Coefficient (Spearman’s Rho)	*p* Value
Rectus femoris muscle thickness on discharge		
Age, years	−0.165	0.280
Body mass index	0.594	<0.001
AIS classification	0.407	0.006
Knee extensor spasticity on admission, MAS	−0.272	0.070
Ankle plantarflexor spasticity on admission, MAS	−0.265	0.079
Onset of mobilization, days	−0.484	0.001
FIM motor subscore on admission	0.361	0.015
LEMS on admission	0.387	0.009
WISCI II on admission	0.366	0.013
Rectus femoris echo intensity on discharge		
Age, years	0.351	0.018
Body mass index	−0.460	0.001
AIS classification	−0.199	0.190
Knee extensor spasticity on admission, MAS	0.193	0.203
Ankle plantarflexor spasticity on admission, MAS	0.190	0.210
Onset of mobilization, days	0.493	0.001
FIM motor subscore on admission	−0.279	0.063
LEMS on admission	−0.270	0.073
WISCI II on admission	−0.199	0.191

AIS: American Spinal Injury Association Impairment Scale; MAS: Modified Ashworth Scale, FIM: Functional Independence Measure; LEMS: Lower Extremity Muscle Score; WISCI II: Walking Index for Spinal Cord Injury II.

**Table 3:** Multivariate analysis of predictors for rectus femoris muscle thickness and echo intensity on discharge.

Variable	Unstandardized Coefficient (B)	95% CI	*p* Value
Rectus femoris muscle thickness on discharge			
Age, years	−0.554	−1.47, 0.369	0.231
Body mass index	4.62	1.77, 7.47	0.002 *
AIS classification	21.67	−11.54, 54.89	0.256
Onset of mobilization, days	−4.97	−9.46, −0.484	0.031 *
FIM motor subscore on admission	−0.160	−1.44, 1.12	0.801
LEMS on admission	−0.077	−1.86, 1.70	0.930
WISCI II on admission	1.54	−1.70, 4.78	0.341
Rectus femoris echo intensity on discharge			
Age, years	0.546	0.126, 0.967	0.012 *
Body mass index	−0.680	−1.98, 0.618	0.295
AIS classification	−0.019	−15.16, 15.12	0.998
Onset of mobilization, days	2.49	0.439, 4.53	0.019 *
FIM motor subscore on admission	−0.072	−0.654, 0.510	0.802
LEMS on admission	−0.063	−0.874, 0.748	0.875
WISCI II on admission	−0.393	−0.197, 0.984	0.503

AIS: American Spinal Injury Association Impairment Scale; CI: Confidence interval; FIM: Functional Independence Measure; LEMS: Lower Extremity Muscle Score; WISCI II: Walking Index for Spinal Cord Injury II. *: <0.05

## Data Availability

The data presented in this study are available on request from the corresponding author.

## Supplementary Materials

The following supporting information can be downloaded at: https://www.mdpi.com/article/10.3390/jcm15041570/s1, Table S1: Strobe.

## Abbreviations

The following abbreviations are used in this manuscript:

AIS	American Spinal Injury Association Impairment Scale
A.U.	Arbitrary Units
BMI	Body Mass Index
CI	Confidence Interval
CSA	Cross-sectional Area
DEXA	Dual-Energy X-ray Absorptiometry
FIM	Functional Independence Measure
LEMS	Lower Extremity Muscle Score
MAS	Modified Ashworth Scale
MRI	Magnetic Resonance Imaging
SCI	Spinal Cord Injury
SD	Standard Deviation
STROBE	Strengthening the Reporting of Observational Studies in Epidemiology
WISCI II	Walking Index for Spinal Cord Injury II
